# Searching and Navigating UniProt Databases

**DOI:** 10.1002/cpz1.700

**Published:** 2023-03

**Authors:** Yvonne C. Lussi, Michele Magrane, Maria J. Martin, Sandra Orchard, The UniProt Consortium

**Affiliations:** 1 European Molecular Biology Laboratory, European Bioinformatics Institute (EMBL-EBI), Cambridge, United Kingdom; 2 Swiss Institute of Bioinformatics, Geneva, Switzerland; 3 Protein Information Resource, Washington, DC

**Keywords:** database, navigation, protein, search, tutorial, UniProt

## Abstract

The Universal Protein Resource (UniProt) is a comprehensive resource for protein sequence and annotation data. The UniProt website receives about 800,000 unique visitors per month and is the primary means to access UniProt. It provides 10 searchable datasets and four main tools. The key UniProt datasets are the UniProt Knowledgebase (UniProtKB), the UniProt Reference Clusters (UniRef), the UniProt Archive (UniParc), and protein sets for completely sequenced genomes (Proteomes). Other supporting datasets include information about proteins that is present in UniProtKB protein entries, such as literature citations, taxonomy, and subcellular locations, among others. This article focuses on how to use UniProt datasets. The first basic protocol describes navigation and searching mechanisms for the UniProt datasets, and two additional protocols build on the first protocol to describe advanced search and query building.

## INTRODUCTION

Understanding protein function is critical to research in many areas of science, such as biology, medicine, and biotechnology. As the number of completely sequenced genomes continues to increase, huge efforts are being made in the research community to understand as much as possible about the proteins encoded by these genomes. This work is generating large amounts of data that are spread across multiple locations, including scientific literature and many biological databases. UniProt, or the Universal Protein Resource, provides an up-to-date, comprehensive body of protein information at a single site.

The UniProt website can be accessed at http://www.uniprot.org. The following three basic protocols describe how you can navigate the site to access datasets and how you can make the most of the search functionality to find your data of interest within these datasets.

## BASIC PROTOCOL 1

### SEARCHING UniProt DATASETS

The UniProt website provides 10 main datasets and four main tools. The key UniProt datasets are the UniProt Knowledgebase (UniProtKB), the UniProt Reference Clusters (UniRef), the UniProt Archive (UniParc), and protein sets for completely sequenced genomes (Proteomes). Supporting datasets include information about proteins that is present in UniProtKB protein entries, such as literature citations, taxonomy, subcellular locations, keywords, cross-referenced databases, and related human diseases. Additional searchable sets include annotation programs used in UniProt and UniProt Help. The four tools that UniProt provides are the “BLAST” sequence search tool, the “Align” multiple sequence alignment tool, the “Peptide Search” tool, and the “ID Mapping” tool, where you can upload lists of identifiers to download corresponding UniProt entries or map them to/from external databases. The following steps describe how to explore and search the different datasets within UniProt.

### Necessary Resources

An up-to-date web browser

**Figure 1:**
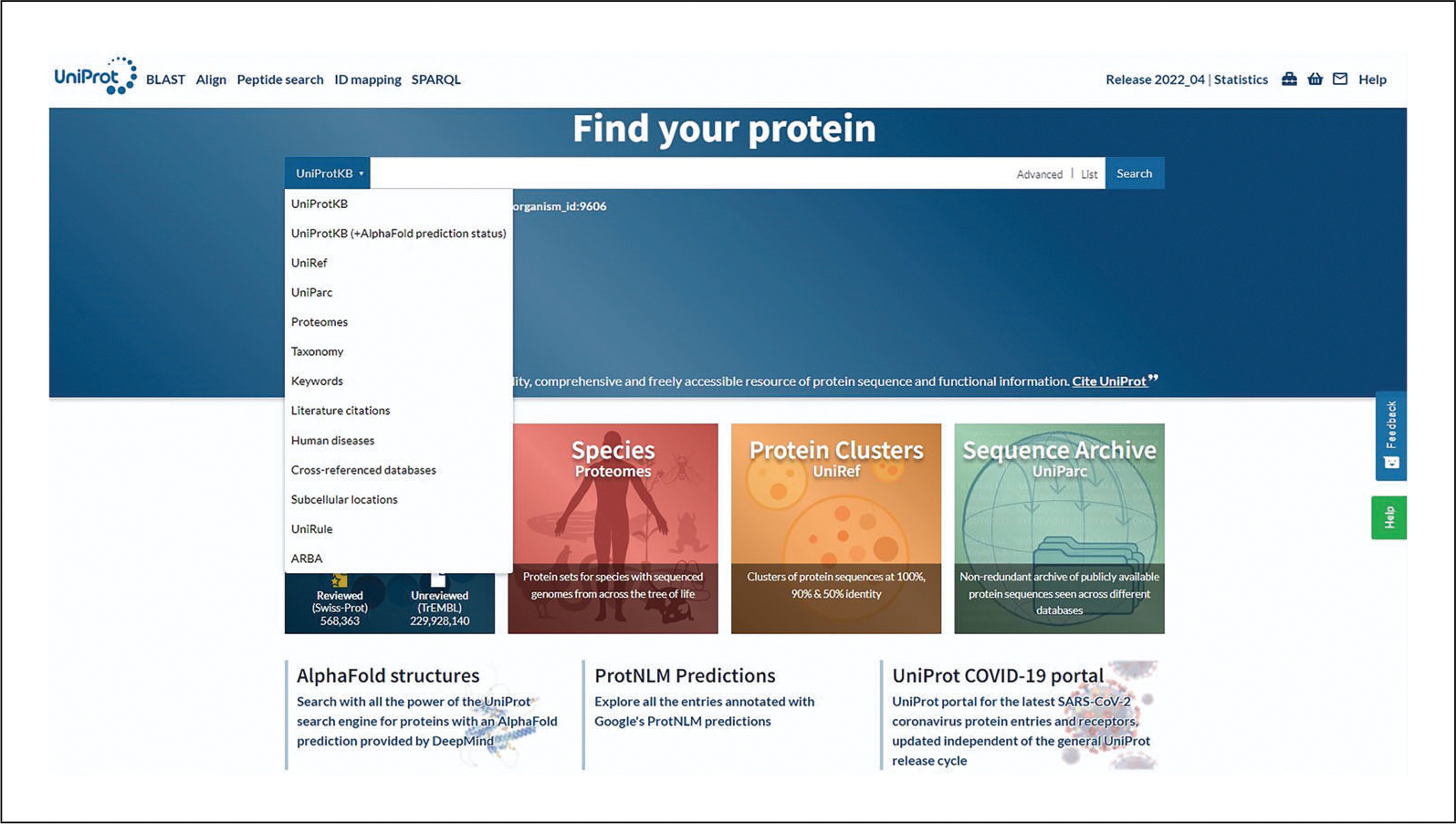
UniProt datasets’ drop-down menu.

**Figure 2:**
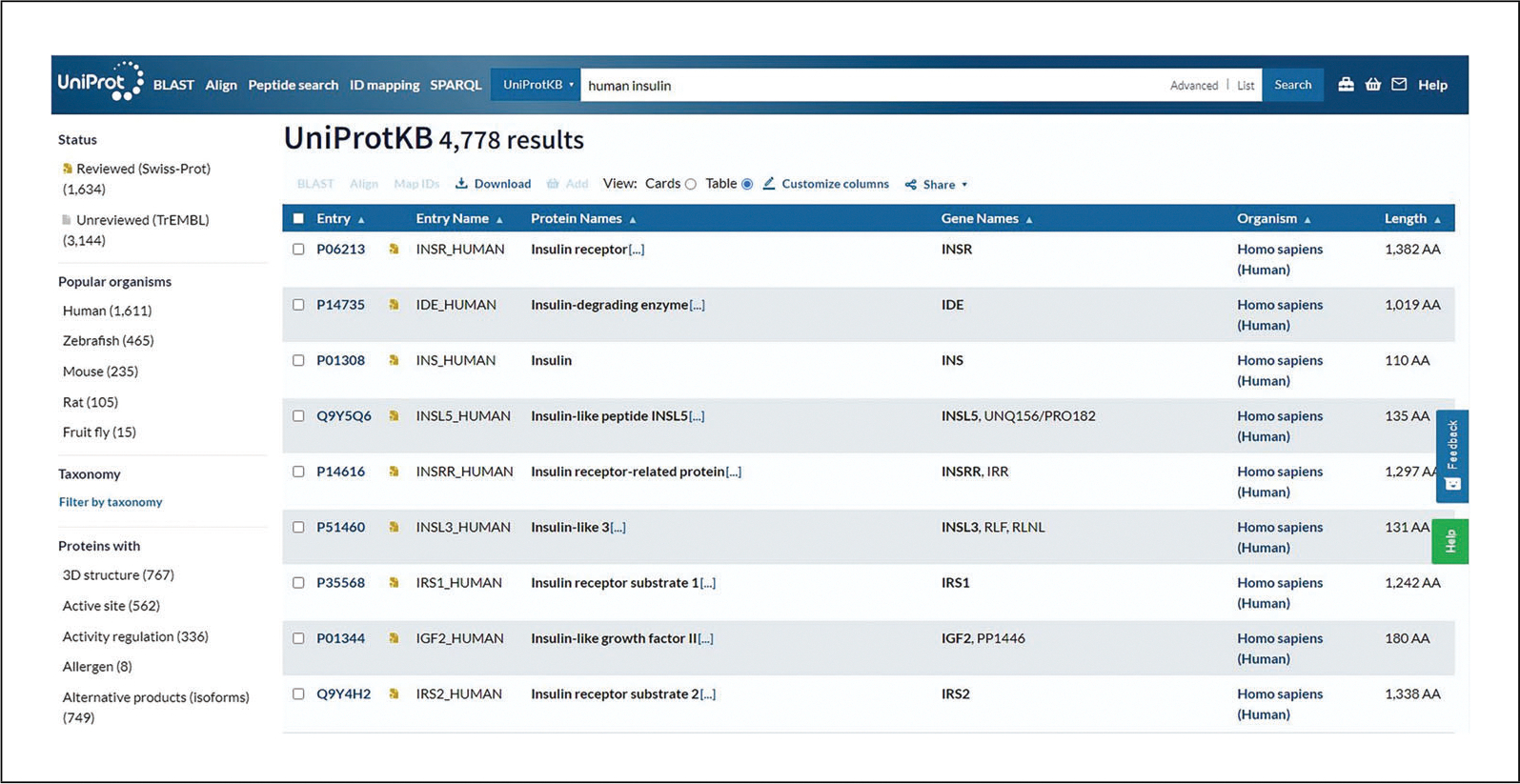
UniProtKB search results page.

**Figure 3:**
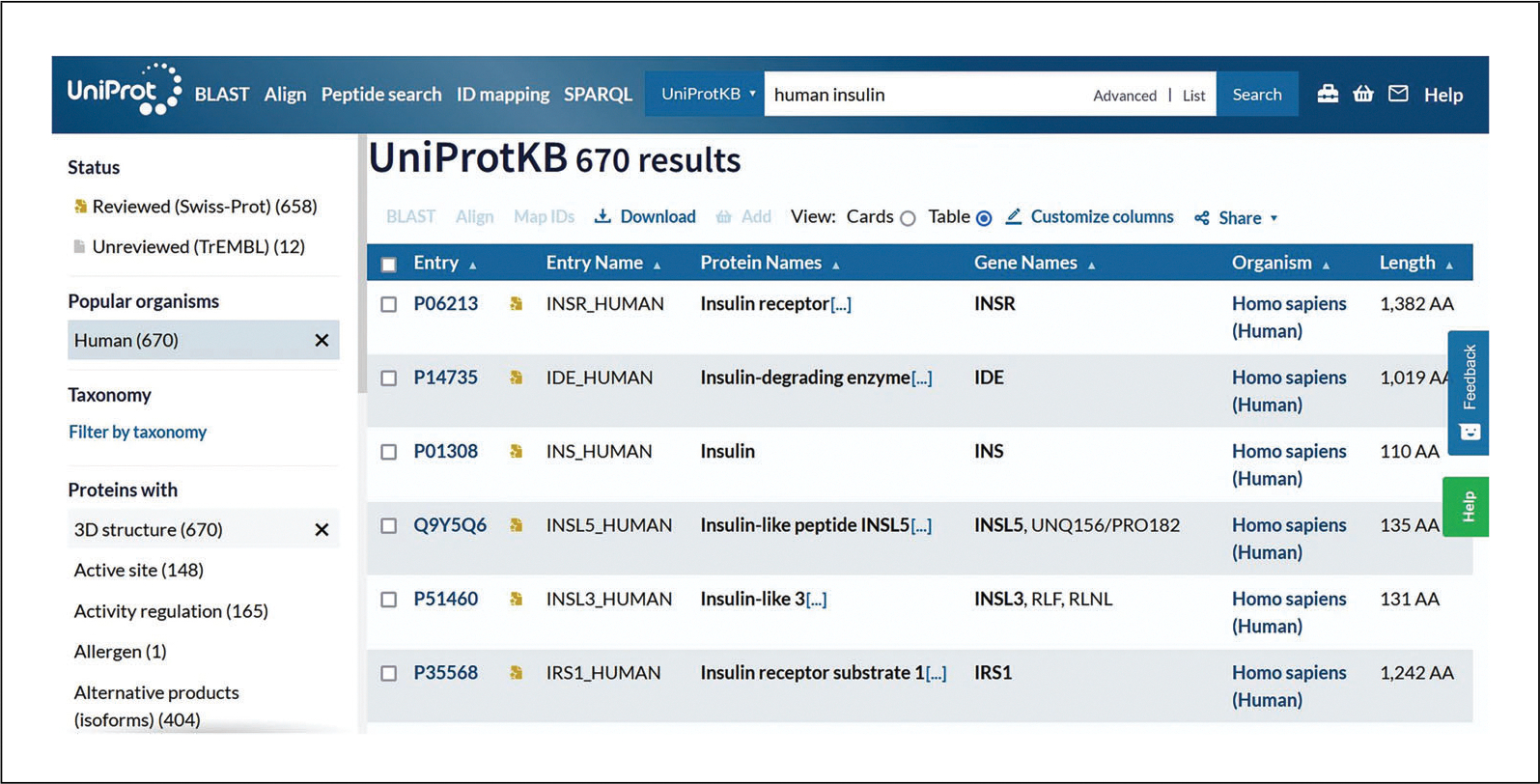
UniProtKB search results with filter applied.

**Figure 4:**
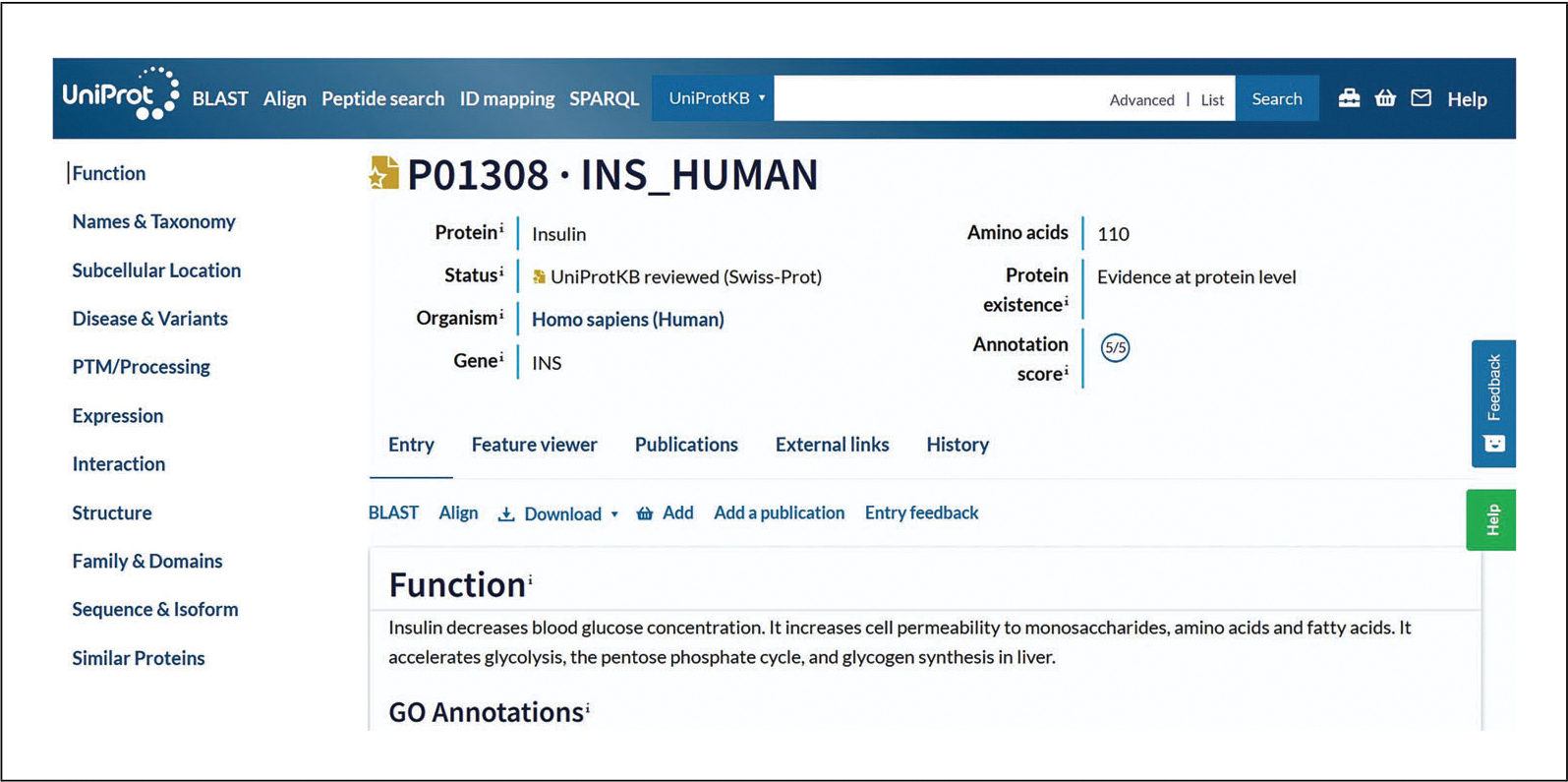
UniProtKB protein entry INS_HUMAN for human insulin.

1. Go the UniProt home page at http://www.uniprot.org using an up-to-date web browser.
2. Click on the drop-down panel to the left of the search box to see all UniProt datasets and select the one that is of interest.
  The drop-down panel to the left of the search box is shown in Figure 1.
  You can also select the main datasets as tiles on the home page. Note that the background color of the drop-down panel changes depending on the dataset in order to help identify the selected dataset.
3. Enter a query in the search box and click the Search button.
  For example, select “UniProtKB” (the default option) and enter “human insulin.” For the full UniProt query syntax, see Table 1.
4. Review the search results page for the dataset.
  The displayed protein entries can be viewed in a “card” or “table” format, selected with the icons at the top of the results page. The results page for the example query in table format is shown in Figure 2. The “card” format gives the user a quick summary of the data present in the entry.
5. Click on the filters on the left to narrow down the results.
  For example, click on “Human” under the “Popular organisms” filter to narrow down the term “human” as the organism. Then, go to the “Proteins with” filters and select the filter “3D structure,” for example. The resulting screen with the selected filters is shown in Figure 3.
6. Click on the accession links under the “Entry” column to view individual protein entries.
  For example, clicking on the link for INS_HUMAN (P01308) will take you to the protein entry for human insulin. The human insulin entry as seen in Figure 4 has a summary at the beginning that displays its protein name, status (i.e., reviewed or unreviewed), organism, gene name, protein length, evidence level, and annotation score. At the top of the entry, the tabs “Entry,” “Feature viewer,” “Publications,” “External links,” and “History” allow exploration of the different sections of the entry. You can use the buttons beneath the summary section to launch tools, view and download the entry in different formats, add it to your basket, add a publication, or give feedback on the entry. When there is an isoform present, you can align the sequences to view similarities and differences. You can explore information and supporting evidence about function, subcellular location, disease, and variants in human entries; phenotypes and variants in non-human entries; and a range of other data categories by clicking on the corresponding buttons in the navigation panel on the left. Further guidance is available within the Guidelines for Understanding Results section.

## BASIC PROTOCOL 2

### ADVANCED SEARCH AND QUERY BUILDING

You can access advanced search options by clicking on “Advanced” to restrict terms to specific fields or to combine multiple terms using Boolean logic. The advanced search provides a query builder, which helps you expand your query. The advanced search translates search terms that have been entered and filters that have been applied into the query builder and can be accessed from all pages.

### Necessary Resources

An up-to-date web browser

**Figure 5:**
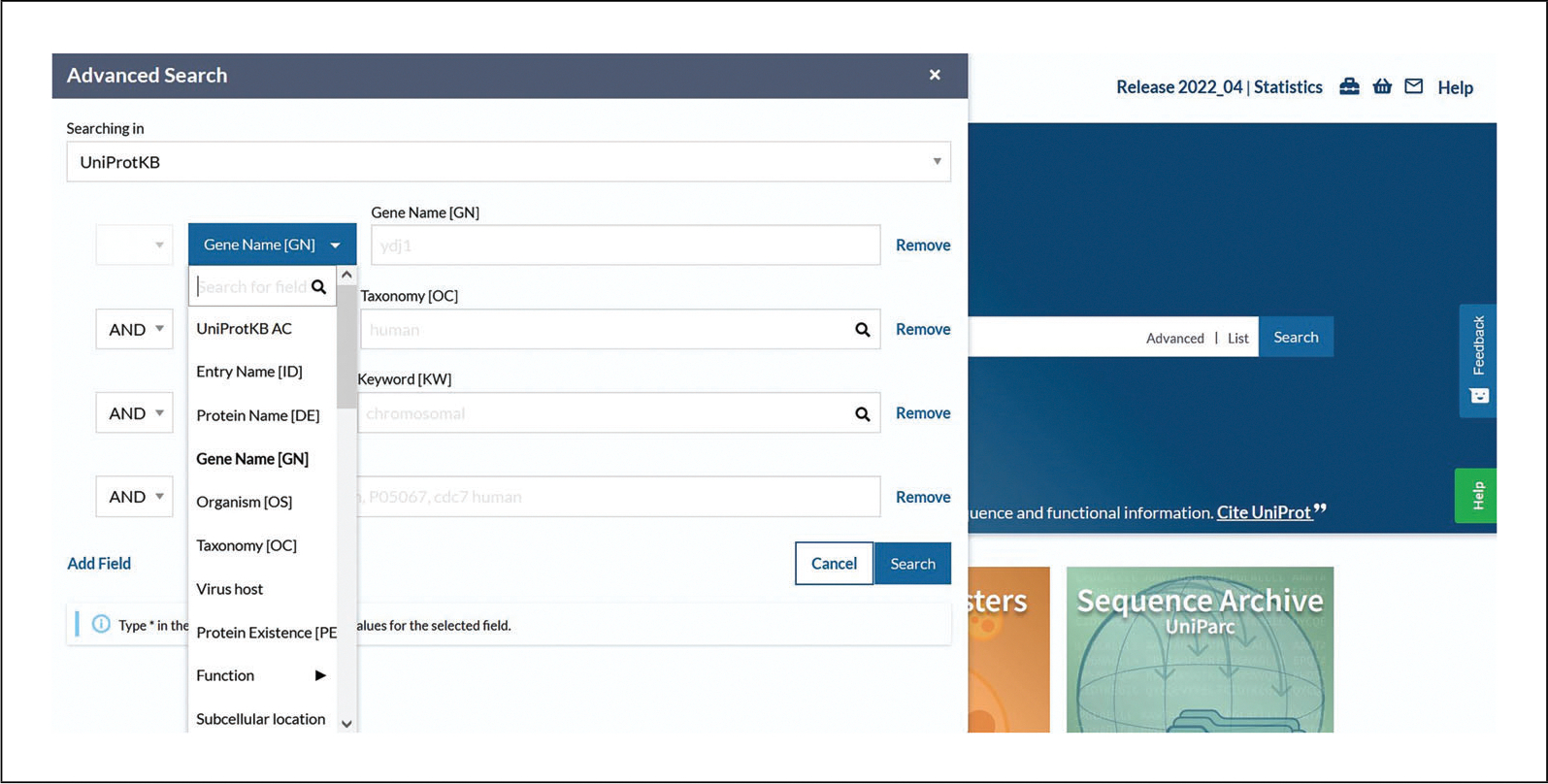
UniProtKB advanced search field types available.

**Figure 6:**
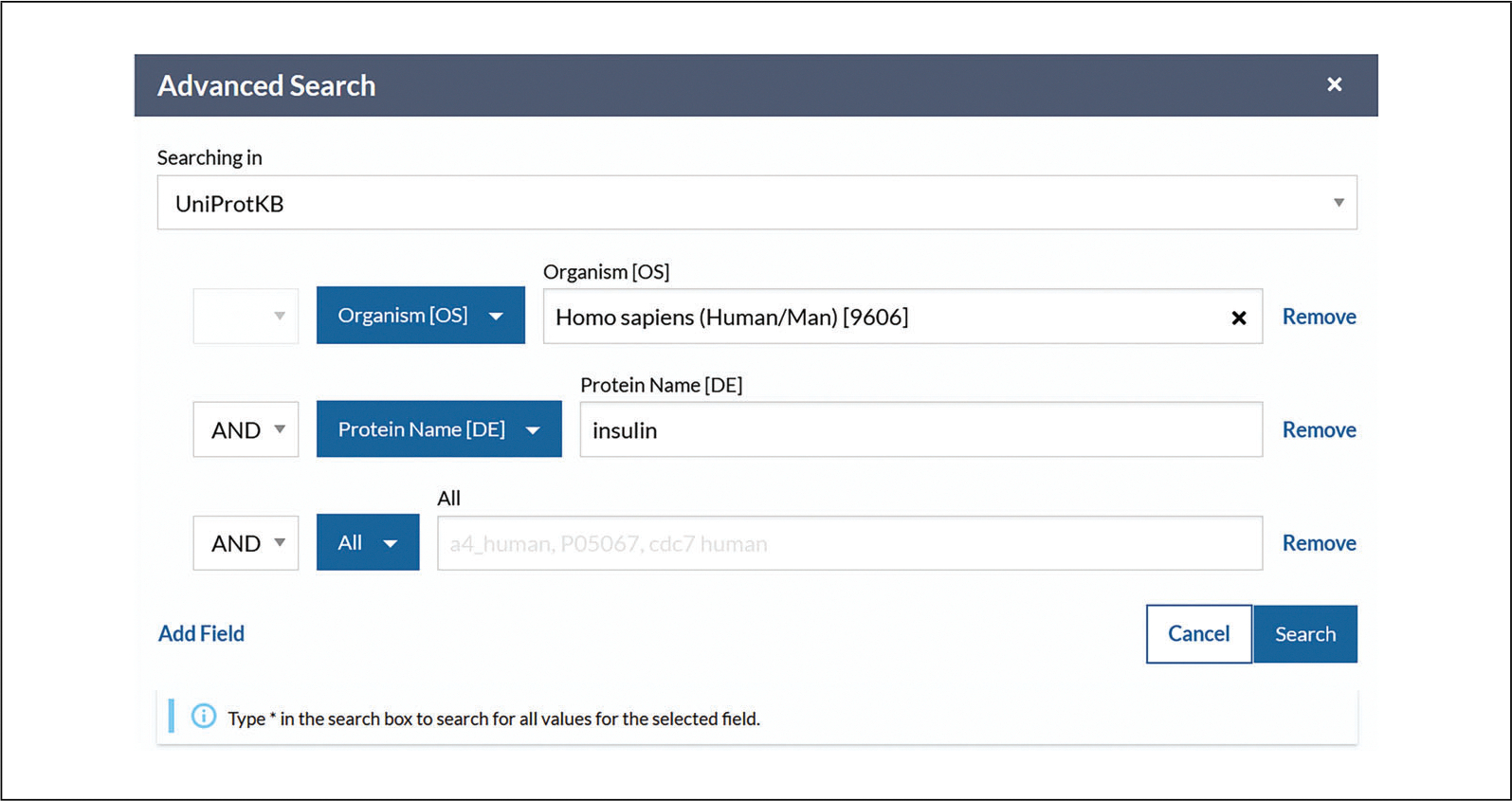
Advanced search example.

**Figure 7:**
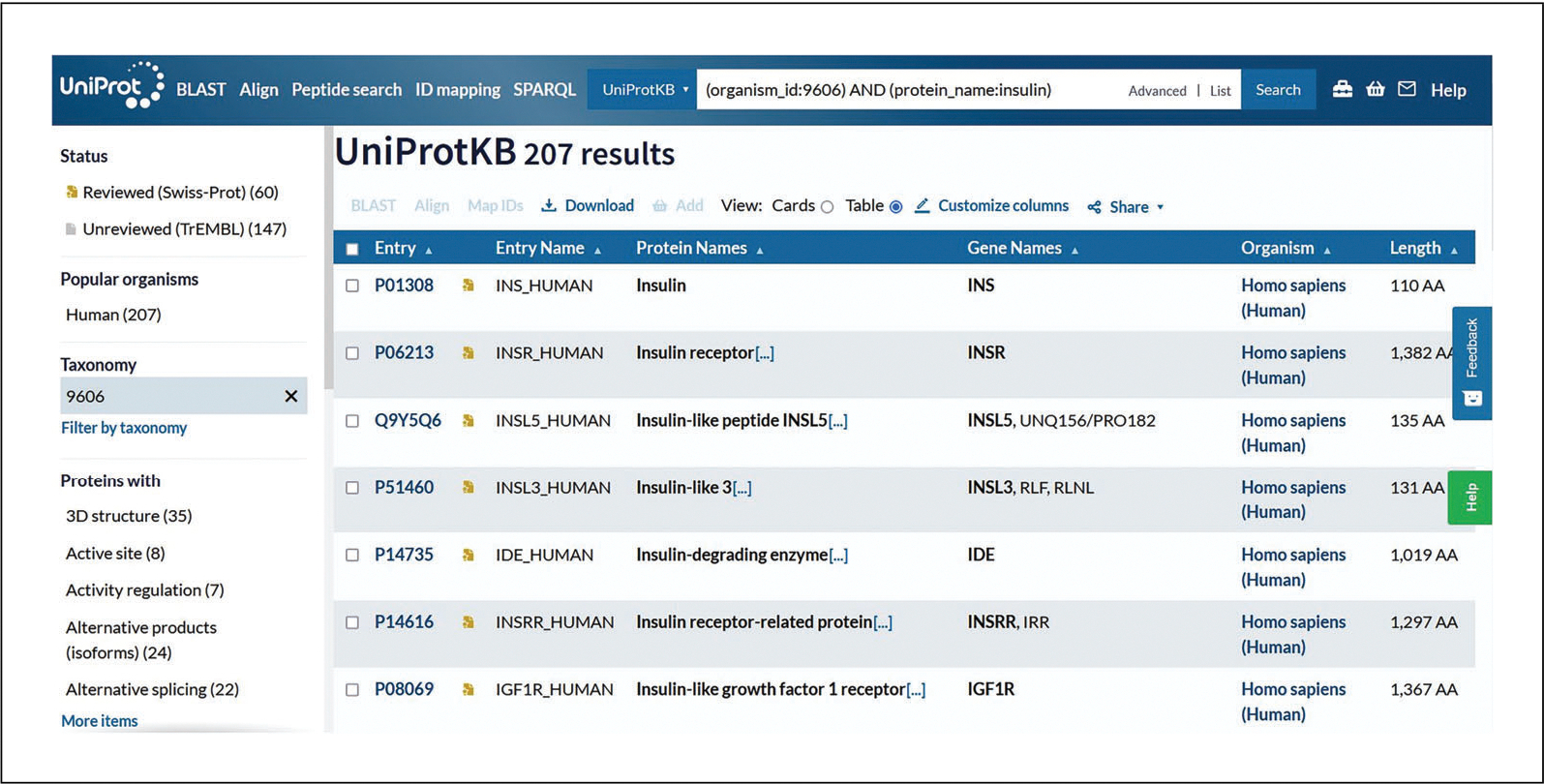
UniProtKB search results through advanced search.

1. Go to the UniProt home page at http://www.uniprot.org using an up-to-date web browser.
2. For help on the advanced search options available, click on the blue “Help” icon in the top right corner or on the green “Help” icon on the right-hand side of each page. Search for “advanced search” to view the advanced search options page, which lists the different options available in the “Advanced Search” menu for UniProtKB.
3. Click on the drop-down menu to the left of the search box and select the dataset.
  The default is UniProtKB.
4. Click on “Advanced” to the right of the search box. Click the drop-down box as seen in Figure 5 to view and choose various field types or type a concept name (e.g., “structure”) and select the most suitable one from the autocompleted suggestions. Click on the “Add field” icon to add more rows and further build the query. If desired, combine the rows by choosing the Boolean operators “And,” “Or,” and/or “Not.” Then, click the search button.
  The advanced search allows you to restrict terms to specific fields in advance or to combine multiple search terms using Boolean logic. The advanced search panel is shown in Figure 6.
  For example, using the drop-down, select the field type “Organism” and enter “Homo sapiens.” Then, under the next drop-down, select the field type “Protein name” and enter “insulin.”
5. View the search results page with the selected parameters reflected in the filters, as in Figure 7.
6. Click on the accession links under the “Entry” column to view individual protein entries.

## BASIC PROTOCOL 3

### ADDING PARAMETERS USING ADVANCED SEARCH

The advanced search can be a powerful tool to narrow down to very specific results. Here, we look at an example where we use various advanced search fields to find all entries in UniProtKB with direct protein sequencing evidence that are encoded in the mitochondrial sequence and have manually entered experimental evidence for function.

### Necessary Resources

An up-to-date web browser

**Figure 8:**
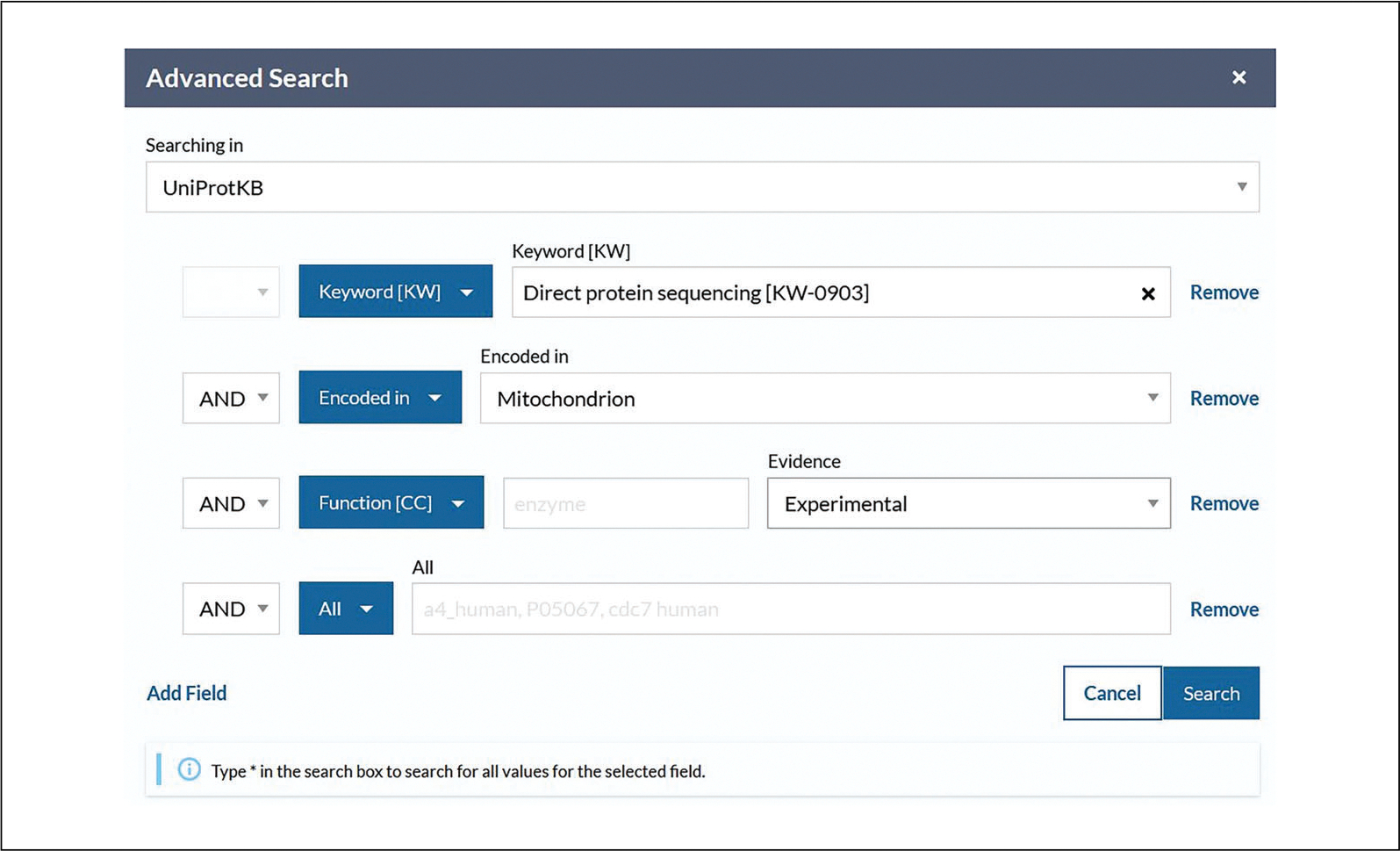
Adding parameters to UniProtKB advanced search.

**Figure 9:**
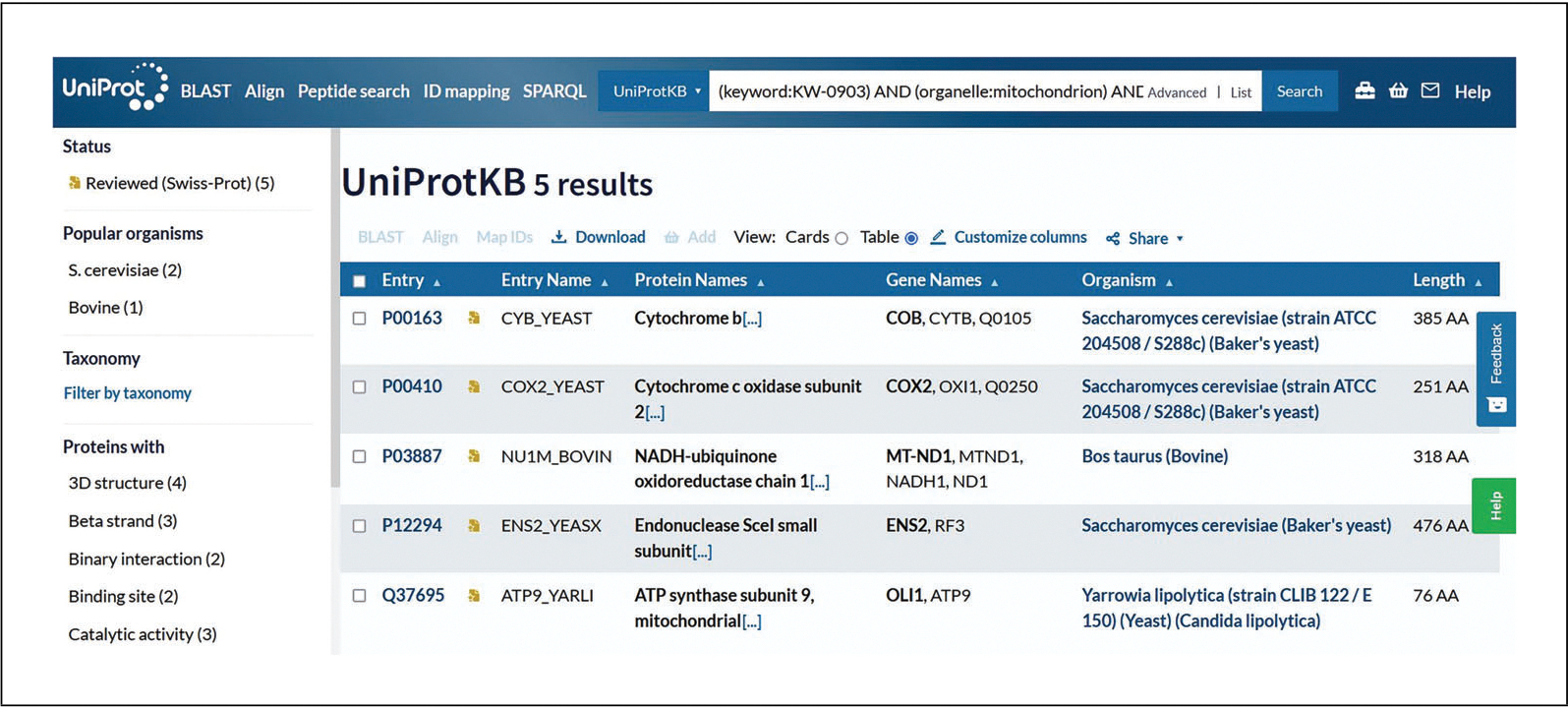
UniProtKB search result for additional advanced search parameters.

1. Go to the UniProt home page at http://www.uniprot.org using an up-to-date web browser.
2. Click on the drop-down to the left of the search box and select “UniProtKB.”
3. Click on “Advanced” to the right of the search box to access advanced search.
4. Click on the drop-down menu box within advanced search, select the option “Keywords,” and type “direct protein sequencing” or select it from the autocomplete drop-down menu after beginning to type.
  For additional exploration, all keywords can be found under the Keywords supporting dataset at http://www.uniprot.org/keywords.
5. Click on the drop-down in the next row and select “Sequence/Encoded in” or select it from the autocomplete drop-down menu after beginning to type. For this example, choose “Mitochondrion” from the drop-down to the right.
6. If required, click on “Add Field” at the bottom of the rows to add another parameter. Click on the new drop-down box and select “Function/Function [CC].” Then, click on the “Evidence” drop-down on the right and select “Experimental,” found under “Manual Assertions.” Verify that the view looks like Figure 8. Leave the input field vacant to include all possible values. Click the search button.
7. View the search results page, in this case with just five results (as of December 2022) that match all inputted parameters, as shown in Figure 9.
8. Click on the entry accession to explore the protein entry in detail.

## GUIDELINES FOR UNDERSTANDING RESULTS

### UniProtKB Search Results

The UniProtKB results page provides filters on the left and the results table on the right. Filters allow you to select results belonging only to the Reviewed (UniProt/Swiss-Prot) or Unreviewed (UniProt/TrEMBL) section or results from just a certain organism using the “Taxonomy” filter. You can also refine your search terms using the “Proteins with” filters. You can view your results according to their taxonomy, protein existence, annotation score, or sequence length.

The results page also provides a row of buttons along the top to access tools, download your results, and add them to your basket. The “Customize columns” button can be used to edit the columns you see to add or remove information. You can select entries to launch BLAST searches, run multiple sequence alignments, and add selected entries to your basket. You can download the entire results table or just your selected entries.

### UniProtKB Protein Entry

**Figure 10:**
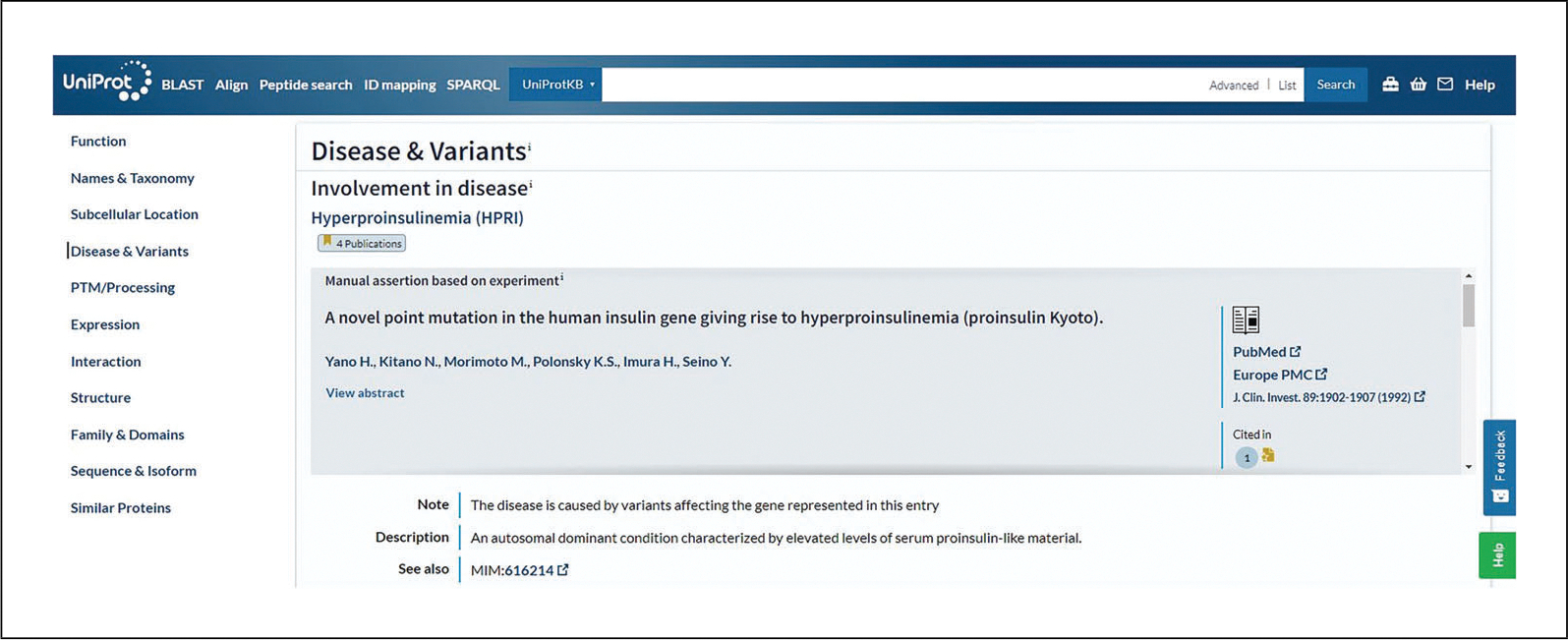
Evidence tags in INS_HUMAN protein entry.

The UniProtKB protein entry page, such as the INS_HUMAN example shown in Figure 4, provides the protein sequence and all the functional annotation related to the protein. This example belongs to the “Reviewed” section, which is expertly annotated by UniProt curators. It has an annotation score of five out of five, indicating a high level of manual annotation. At the top of the entry, the tabs “Feature viewer,” “Publications,” “External links,” and “History” allow for additional ways to explore the data in the entry. You can view the evidence of annotated information using evidence tags. For example, the “Disease & Variants” section presents a “4 Publications” evidence tag (as of December 2022) for involvement of the protein in hyperinsulinemia, which can be opened to view a summary of the publications cited, as seen in Figure 10. Evidence tags are color coded such that gold indicates a manually entered assertion and silver indicates an automatically entered assertion.

### Aligning Isoforms

**Figure 11:**
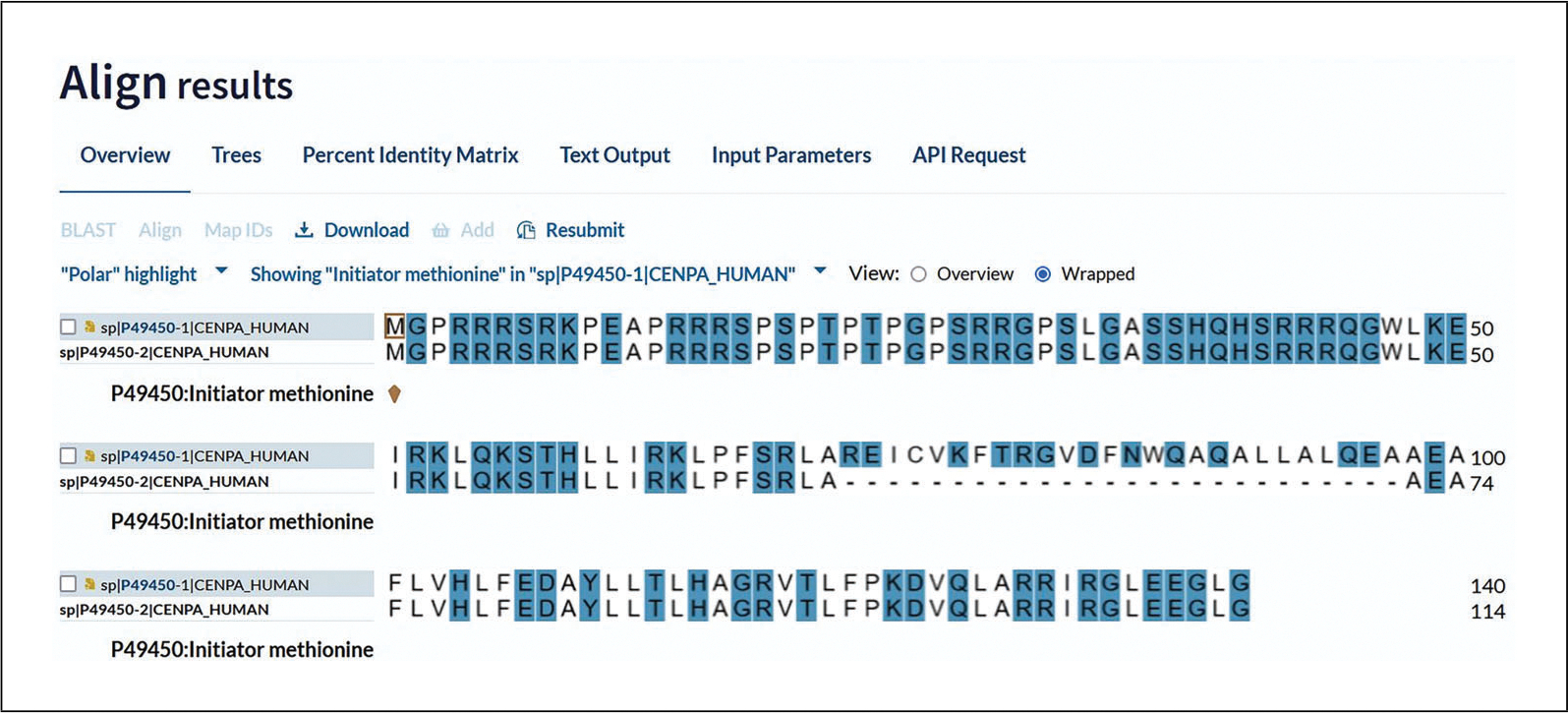
Highlighted alignment for CENPA_HUMAN sequences.

The entry page also provides various buttons for tools and formats and for adding the entry to your basket under the summary section at the top of the page. There are also other tools available for sequences in the “Sequence & Isoform” section. When there are isoforms present, you can align them using the “Align” button that is at the top of the page and also within the “Sequence & Isoform” section. The results show the sequence alignment including gaps, as shown in Figure 11 for human CENPA (P49450). There is a “Select annotation” drop-down menu at the top that allows you to highlight sequence feature annotations to view them in the alignment. Sequence annotations are mapped to the main canonical sequence, so some features may not be available for all isoforms. You can compare highlighted regions across the aligned isoform sequences to view conserved areas and differences. The “Highlight properties” drop-down menu at the top left allows you to highlight amino acids in the alignment according to their properties. For example, you can view all the polar amino acids in the aligned sequences by clicking on “Polar” as shown in Figure 11.

### Add a Publication

**Figure 12:**
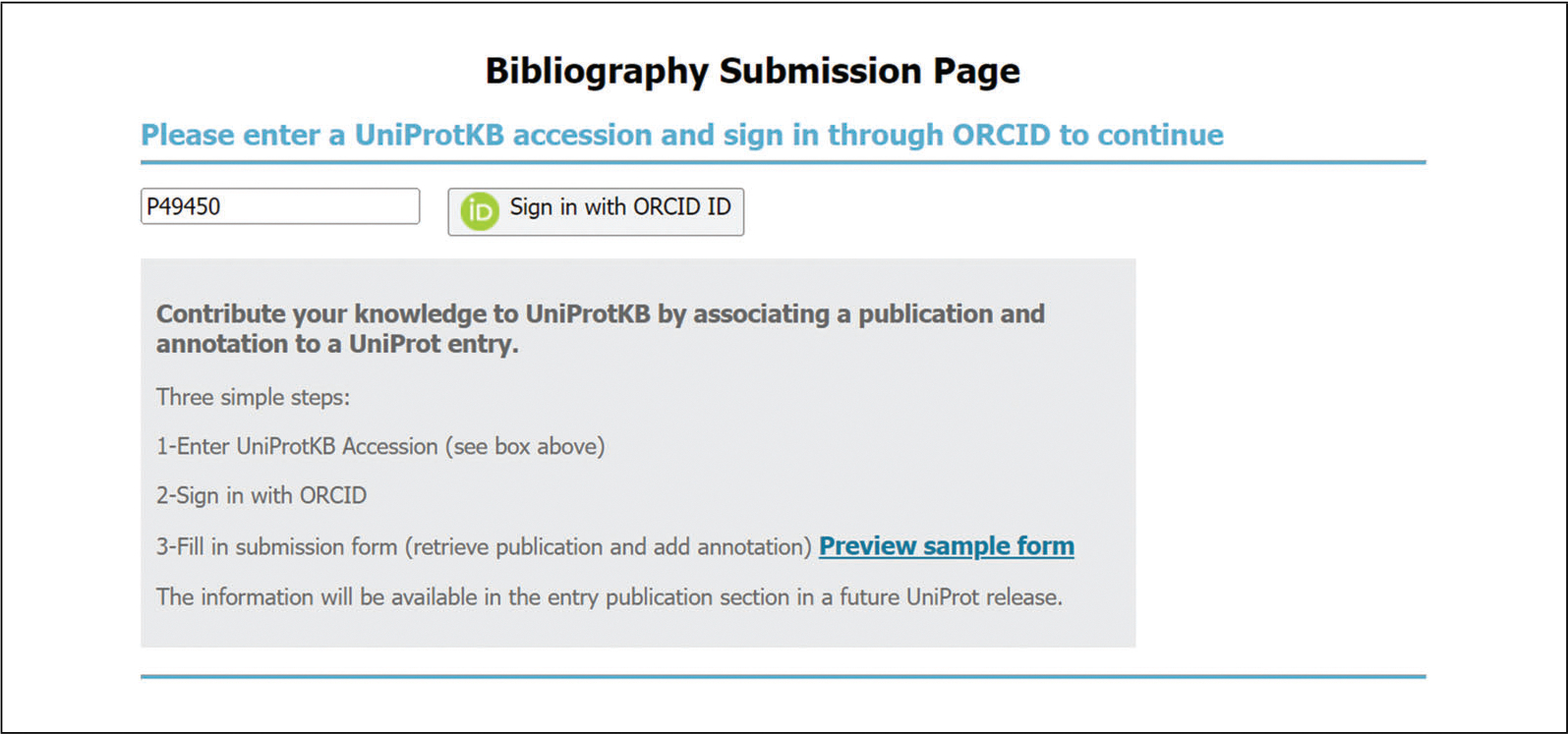
UniProtKB Bibliography Submission Page.

UniProt users have the possibility of submitting their own publication or adding an article of interest to a UniProtKB entry via the community submission pipeline. At the top of each protein entry, there is an “Add a publication” link, which will take you to the Bibliography Submission Page as shown in Figure 12. Along with the publication, optional basic annotations can be provided by selecting the topics relevant to each paper from a controlled list and/or adding short statements about protein name, function, and disease, as described in the publication. We ask you to supply your ORCID, a researcher personal ID, which is used both to validate that the submission is genuine and to give you credit for your work. Publications submitted in this manner are included, after a synchronization delay, in the list of publications for the relevant entry and will soon be displayed as annotation for the protein entry.

## COMMENTARY

### Background Information

UniProt aids scientific discovery by collecting, interpreting, and organizing information so that it is easy to access and use. It saves researchers countless hours of work in monitoring and collecting this information themselves. The UniProt Knowledgebase (UniProtKB) is the central hub for the collection of functional information and other rich annotations on proteins. It is further divided into the Reviewed (UniProt/Swiss-Prot), expertly annotated section and the Unreviewed (UniProt/TrEMBL), automatically annotated section. The UniProt Archive (UniParc) is a non-redundant archive containing all the publicly available protein sequences in the world. The UniProt Reference Clusters (UniRef) provide clustered sets of sequences from UniProtKB (including isoforms) and selected UniParc entries. UniRef reduces redundancy and provides complete coverage of the sequence space at three levels of sequence identity (i.e., 100%, 90%, and 50% identity). The Proteomes dataset provides protein sets for organisms with completely sequenced genomes. Supporting datasets are a collection of meta-information about proteins in UniProtKB entries such as literature citations, taxonomy, subcellular locations, keywords, cross-referenced databases, and related diseases.

The UniProt website provides powerful searching and filtering features to help users find the exact data they are interested in. This is further enhanced by a flexible and effective advanced search system, which allows users to define their search terms and build their queries. UniProt provides training material through the European Bioinformatics Institute’s online training portal, including a quick tour (http://www.ebi.ac.uk/training/online/course/uniprot-quick-tourversion-0) and a detailed course (http://www.ebi.ac.uk/training/online/course/uniprot-exploring-protein-sequence-and-functional). UniProt also provides short video tutorials embedded in the website and also available on a YouTube channel at https://www.youtube.com/uniprotvideos.

## Figures and Tables

**Table 1:** UniProt Query Syntax

Query^a^	Description

**human antigen** **human AND antigen** **human && antigen**	All entries containing both terms.
**“human antigen”**	All entries containing both terms in the exact order.
**human -antigen** **human NOT antigen** **human !antigen**	All entries containing the term “human” but not “antigen.”
**human OR mouse** **human || mouse**	All entries containing either term.
**antigen AND (human OR mouse)**	Using parentheses to override Boolean precedence rules.
anti*	All entries containing terms starting with “anti.” Asterisks can also be used at the beginning and within terms. Note that starting with an asterisk or a single letter followed by an asterisk can slow down queries considerably.
author:Tiger*	Citations that have an author whose name starts with “Tiger.” To search in a specific field of a dataset, you must prefix your search term with the field name and a colon. To discover what fields can be queried explicitly, observe the query hints that are shown after submitting a query or use the query builder (see Basic Protocol 2).
length:[100 TO *]	All entries with a sequence of at least 100 amino acids.
**(lit_author:Arai) AND (lit_author:Chung)**	All entries with publications authored by either author.

a To use characters that have a special meaning in the query syntax literally in your query, you must escape them with a backslash, e.g., use “gene:L\(1\)2CB” to search for the gene name L(1)2CB. The current list of special characters is as follows: + - && || ! () {} [] ^ " ~ * ?: \\

## Data Availability

Data sharing is not applicable to this article as no new data were created or analyzed in this study.

